# Dr. Harry F. Seaman

**Published:** 1914-06

**Authors:** 


Dr. Harry F. Seaman, Geneva, 1871; died at his home in Alton N. Y., February 16, aged 71.

